# miR-124-3p sabotages lncRNA MALAT1 stability to repress chondrocyte pyroptosis and relieve cartilage injury in osteoarthritis

**DOI:** 10.1186/s13018-022-03334-8

**Published:** 2022-10-15

**Authors:** Rigbat Rozi, Yubo Zhou, Kai Rong, Pingbo Chen

**Affiliations:** Department of Fourth Orthopedics, Traditional Chinese Medicine Hospital, Affiliated to Xinjiang Medical University, No. 116, Huanghe Road, Ürümqi, 830000 Xinjiang People’s Republic of China

**Keywords:** Osteoarthritis, microRNA-124-3p, LncRNA MALAT1, Pyroptosis, LncRNA stability, Chondrocytes

## Abstract

**Background:**

Osteoarthritis (OA) is a prevalent inflammatory joint disorder. microRNAs (miRNAs) are increasingly involved in OA.

**Aim:**

Our study is proposed to clarify the role of miR-124-3p in chondrocyte pyroptosis and cartilage injury in OA.

**Methods:**

OA mouse model was established via the treatment of destabilization of the medial meniscus (DMM), and the in vitro cell model was also established as mouse chondrocytes were induced by lipopolysaccharide (LPS). Mouse cartilage injury was assessed using safranin-O-fast green staining, hematoxylin–eosin staining, and OARSI grading method. Expressions of miR-124-3p, MALAT1, KLF5, and CXCL11 were determined. Cartilage injury (MMP-13, osteocalcin), inflammation (IL-6, IL-2, TNF-, IL-1β, and IL-18)- and pyroptosis-related factors (Cleaved Caspase-1 and GSDMD-N) levels were detected. Mechanically, MALAT1 subcellular localization was confirmed. The binding relationships of miR-124-3p and MALAT1 and MALAT1 and KLF5 were verified. MALAT1 half-life period was detected. Then, miR-124-3p was overexpressed using agomiR-124-3p to perform the rescue experiments with oe-MALAT1 or oe-CXCL11.

**Results:**

miR-124-3p was downregulated in DMM mice and LPS-induced chondrocytes where cartilage injury, and increased levels of inflammation- and pyroptosis-related factors were found. miR-124-3p overexpression relieved cartilage injury and repressed chondrocyte pyroptosis. miR-124-3p bounds to MALAT1 to downregulate its stability and expression, and MALAT1 bounds to KLF5 to enhance CXCL11 transcription. Overexpression of MALAT1 or CXCL11 annulled the repressive function of miR-124-3p in chondrocyte pyroptosis.

**Conclusion:**

miR-124-3p reduced MALAT1 stability and inhibited the binding of MALAT1 and KLF5 to downregulate CXCL11, thereby suppressing chondrocyte pyroptosis and cartilage injury in OA.

## Background

Osteoarthritis (OA) is defined as a kind of degenerative joint disorder with the traits, such as long-term pain and organic dysfunction, and is becoming epidemic worldwide along with the growing rates of obesity and aging [1]. OA may occur in all joints and is prevalent due to overweight, excessive smoking, poor nutrition, occupational injury, physical activities, aging, joint disorganization, hereditary factor, and unhealthy lifestyle [2]. To date, joint replacement is the only effective strategy for OA, but its side effect might induce unsatisfactory clinic consequences and even discounted living quality [3]. Primarily, OA brings about severe cartilage injury and a high disability rate for subjects with OA [4]. Meanwhile, inflammatory factor release is observed in different sites of OA joints [5]. Furthermore, intensive and persistent pyroptosis in soft tissues might induce cartilage injury, augmented anguish, or hypersensitivity when subjected to injurious stimulation and eventually secrete cytokines to strengthen pain from OA [6]. Against this background, the management to alleviate chondrocyte pyroptosis and repair cartilage injury is warranted for OA therapy.

microRNAs (miRNAs) are modulators of gene translation and metabolic activities of OA chondrocytes [7]. Besides, given their role in regulating gene expression and physiopathology of OA, they can be also used as makers of the disease and therapeutic targets [8]. miRNAs mediate the cellular biological behaviors including pyroptosis, to affect the pathogenesis and development of human disorders [9]. Significantly, miR-124-3p expression is decreased in OA [10]. miR-124-3p strengthens the anti-inflammatory efficacy of the candidate medicine in suppressing cartilage injury and protecting joint structure [11]. Moreover, miR-124-3p reverses the increasing levels of pyroptosis indicators [12]. Primarily, miRNAs influence the molecular modification and gene phenotypes of long non-coding RNAs (lncRNAs) to control the stability and expression of lncRNAs in different biological behaviors [13]. LncRNAs, a family of intensively researched RNAs, are also candidate biomarkers for OA by mediating OA development and providing diagnostic and prognostic insights for OA [14]. LncRNA metastasis-associated lung adenocarcinoma transcript 1 (MALAT1) is accumulated in individuals assaulted by OA and exacerbates inflammatory reactions and cartilage injury [15]. On the other hand, MALAT1 can stimulate the release of inflammatory cytokines and exert pro-pyroptotic influences on pathological changes [16]. LncRNAs are involved in human diseases by interacting with transcription factors to modulate molecular activities [17]. Consistently, MALAT1 can positively regulate the level of transcription factor Krüppel-like factor 5 (KLF5) to enhance cellular damage in diabetic nephropathy [18]. KLF5 intensifies inflammatory symptoms, chondrocyte apoptosis, and cartilage degradation in OA [19]. Taking the above-listed evidence into consideration, we hypothesize that miR-124-3p might mediate chondrocyte pyroptosis and cartilage injury in OA via the modulation of MALAT1 and its downstream pathway.

## Materials and methods

### Ethics statement

This study was approved and supervised by the ethics committee of Traditional Chinese Medicine Hospital Affiliated to Xinjiang Medical University. The protocol was also approved by the Institutional Animal Care and Use Committee of Traditional Chinese Medicine Hospital Affiliated to Xinjiang Medical University and the *Guidelines for the Care and Use of Laboratory Animals* provisions of administration and usage of laboratory animals [20]. Significant efforts were made to minimize both the number of animals and their respective suffering.

### Laboratory animals

Forty-eight male C57BL/6 mice [12 weeks old, Laboratory Animals Monitoring Institute, Guangzhou, Guangdong, China, SCXK (Guangdong) 2018-0044] were kept in 12-h light–dark cycles with constant temperature (22 ± 2 °C) and relative humidity (60%) for 1 week before the OA mouse model was established.

### Animal model establishment

After a week of adjustable feeding, mice were subject to destabilization of the medial meniscus (DMM) to establish the OA mouse model. The mice were divided into the DMM group and the sham group. Mice in the DMM group were anaesthetized through an intraperitoneal injection of 2% pentobarbital sodium (40 mg/kg) and then subject to the right knee articular capsule incision at the medial patellar tendon, with the medial meniscotibial ligament (MMTL) transected using microsurgical scissors to establish the DMM model. Mice were given fluid resuscitation (1 mL/mouse sterile saline via subcutaneous injection) immediately after surgery. Mice in the sham group were treated in the same manner as the DMM group except for the transfection of MMTL. All mice had access to adequate food and water after surgery.

After 48 h of the DMM surgery, agomiR-124-3p was injected into the joint of the OA mice, with agomiR-NC as the negative control (NC).

### Blood collection and tissue section

After 8 weeks of surgery, 1 mL of whole blood was collected by removing the eyeball. Next, the blood was rested for 40 min and centrifuged at 1000 × *g* for 10 min to separate the serum, which was preserved in a − 80 °C refrigerator for further experiments. After blood collection, mice were euthanized via an injection of 200 mg/kg sodium pentobarbital, and knee joint tissues were collected from each group. Six pairs of knee tissues from each group were prepared into tissue sections. Briefly, knee tissues were fixed in 4% paraformaldehyde for 24 h, decalcified in 10% ethylenediaminetetraacetic acid for 8 weeks, dehydrated in gradient ethanol, paraffin-embedded, and made into sections (5 μm) for subsequent experiments. Six pairs of knee tissues from each group were prepared into tissue homogenates for the following experiments.

### Hematoxylin–eosin (H&E) and safranin-O (S–O) staining

The prepared 5-μm sections were stained with H&E or S–O for the observation of knee joint structure using an optical microscope (Olympus Optical Co., Ltd, Tokyo, Japan). OA cartilage degeneration was assessed by 3 independent researchers according to the Osteoarthritis Research Society International (OARSI) grading method [21]. The grading ranges from Grade 0 (normal) to Grade 6 as intact cartilage and surface, Grade 0; intact surface, Grade 1; surface incontinuity, Grade 2; vertical fracture, Grade 3; erosion, Grade 4; denudation, Grade 5; and deformation, Grade 6.

### Cell culture and treatment

Primary mouse chondrocytes were isolated from neonatal C57BL/6 male mice [5 days old, Laboratory Animals Monitoring Institute, SCXK (Guangdong) 2018-0044] via the collagenase digestion method according to a previous study [22]. Briefly, mice were euthanized via the intraperitoneal injection of 200 mg/kg sodium pentobarbital to remove articular cartilage, which was detached using 3 mg/mL collagenase D at 37 °C with 5% CO_2_ for 90 min, detached using 0.5 mg/mL collagenase D 37 °C overnight, and then centrifuged at 400 × *g* for 10 min to discard the supernatant. The cell precipitate was resuspended in Dulbecco’s modified Eagle medium containing 100 IU/mL penicillin and 0.1 mg/mL streptomycin (Gibco Company, Grand Island, NY, USA). Next, chondrocytes (8 × 10^3^ cells/cm^2^) were seeded into culture dishes, and the medium was refreshed after 2 d. The isolated chondrocytes were confluent after 6–7 d. The cells of passage 2 were employed for subsequent analysis.

agomiR-124-3p, agomiR-NC, overexpression (oe)-MALAT1, oe-CXCL11, oe-NC, small interfering (si) RNAs of KLF5 (si-KLF5-1 and si-KLF5-2) and si-NC (all from GenePharma Co, Ltd, Shanghai, China) were transfected into cells following the instructions of Lipofectamine 2000 (Invitrogen Inc., Carlsbad, CA, USA). Subsequently, cells were preserved in the medium for 48 h for subsequent analysis.

### Cell counting kit-8 (CCK-8) method

The CCK-8 kit (Keygen Biotech Co., Ltd, Nanjing, Jiangsu, China) was employed to assess chondrocyte activity. Chondrocytes (5 × 10^3^ cells/cm^2^) were seeded in 96-well plates, and 10 μL CCK-8 reagent was supplemented into each well, followed by the culture at 37 °C for 2 h. Optical density value at the wavelength of 450 nm was determined. Each procedure was repeated 3 times.

### Reverse transcription quantitative polymerase chain reaction (RT-qPCR)

The total RNA was extracted using the TRIzol reagent (Invitrogen) and RNA was reverse-transcribed into cDNA via the RT kits (R&D Systems Inc., Minneapolis, MN, USA), and the purity and concentration of RNA were determined using Nano-Drop ND-1000 (NanoDrop Technologies Inc., Wilmington, DE, USA). U6 served as the internal reference of miR-124-3p, and glyceraldehyde-3-phosphate dehydrogenase (GAPDH) served as the internal reference of other genes. cDNA was screened using the QuantiTect SYBR Green PCR kits (Thermo Fisher, Shanghai, China) for RT-qPCR. All samples were employed for RT-qPCR via the ABI7500 qPCR system (Applied Biosystems, Inc., Carlsbad, CA, USA). The RT-qPCR primers are seen in Table 1. The relative expressions of genes were calculated using the 2^−ΔΔCt^ method. Each procedure was repeated 3 times.

**Table 1:** Primer sequence of RT-qPCR

	Forward primer (5′-3′)	Reverse primer (5′-3′)
miR-124-3p	ACAGGCTAAGGCTCCCAGTGAA	CGCAGGGTCCGAGGTATTC
MALAT1	GCATTCAGGCAGCGAGAGCAGAGC	GAGATATTTAGTTTTTATTTCATA
KLF5	CCCACGCGGGTGCTGACCATGAGC	GTTCTGGTGGCGCTTCATGTGCAG
CXCL11	AACAGGAAGGTCACAGCCATAGCC	CATGTTTTGACGCCTTAAAAAATT
GAPDH	CTGCCCTTACCCCGGGGTCCCAGC	TTACTCCTTGGAGGCCATGTAGGC
U6	GTGCTCGCTTCGGCAGCACATATA	AATATGGAACGCTTCACGAATT

*RT-qPCR* reverse transcription-quantitative polymerase chain reaction; *miR* microRNA; *MALAT1* metastasis-associated lung adenocarcinoma transcript 1; *KLF5* Krüppel-like factor 5; *CXCL11* C-X-C motif chemokine 11; *GAPDH* glyceraldehyde-3-phosphate dehydrogenase

### Western blot analysis

The total proteins were extracted from tissues or cells using PRO-PREPTM protein extraction buffer (iNtRON Biotechnology, Seongnam, South Korea), and the protein concentration was measured using the Bio-Rad protein assay kits (R&D). Protein samples were separated through sodium dodecyl sulfate–polyacrylamide gel electrophoresis and then transferred onto polyvinylidene fluoride membranes, followed by membrane culture with Tris-buffered saline with Tween 20 (TBST) containing 5% skim milk (BD Biosciences, Sparks, MD, USA) and incubation with rabbit monoclonal antibody Cleaved Caspase-1 (89332, 1:1000, Cell Signaling Technology, Beverly, MA, USA), rabbit monoclonal antibody gasdermin D N-terminal (GSDMD-N, ab8245, 1: 500, Abcam Inc., Cambridge, MA, USA) and rabbit polyclonal antibody GAPDH (ab9485, 1: 2500, Abcam) overnight. Next, the membranes were washed, incubated with goat anti-rabbit immunoglobulin G (IgG, ab6721, 1: 2000, Abcam), and then subjected to 3 washes with TBST. Bands were developed using enhanced chemiluminescence (Thermo Scientific, Pierce, Rockford, IL, USA), and the results were analyzed by the Image J software (National Institutes of Health, Bethesda, MD, USA), with GAPDH as the internal reference. Each experiment was conducted 3 times.

### Enzyme-linked immunosorbent assay (ELISA)

Osteocalcin (OC) and matrix metalloproteinase (MMP)-13 levels in the mouse serum were detected to evaluate mouse cartilage injury. The levels of interleukin (IL)-6, IL-2, and tumor necrosis factor-α (TNF-α) were assessed to analyze mouse inflammation, and variances of IL‐1β and IL‐18 in mouse serum and cell suspension were detected to evaluate cell pyroptosis under the instructions of the kits (R&D).

### Bioinformatics analysis

The binding relation between miR-124-3p and MALAT1 was predicted via StarBase (http://starbase.sysu.edu.cn/) [23]. The subcellular localization of MALAT1 was predicted through the LncATLAS website (http://lncatlas.crg.eu/) [24]. The binding relations between MALAT1 and KLF5 and between KLF5 and CXCL11 were predicted via the RNAInter database (http://www.rna-society.org/rnainter/) [25].

### Fractionation of nuclear and cytoplasmic RNA

The PARIS™ kit (R&D) was employed for the fractionation of cytoplasm and nucleus in compliance with the manufacturers’ instructions. Cells (1 × 10^7^ cells/well) were collected, resuspended in cell fractionation buffer for the separation of cytoplasm and nucleus, and then placed on ice for 10 min, and centrifuged at 4 °C and 5000 × *g* for 30 s. Next, following the instructions of the cell fractionation buffer, the supernatant and nuclear precipitation were separated and preserved for RNA extraction.

### Dual-luciferase reporter gene assay

Wild type (WT) and mutant type (MUT) of MALAT1 fragments containing the binding sites of miR-124-3p were constructed into the pmirGLO-reporter vector (Beijing Huayueyang Biotechnology, Beijing, China). The constructed luciferase reporter plasmids were co-transfected with agomiR-NC or agomiR-124-3p into mouse chondrocytes. After 48 h, cells were collected, lysed, and the luciferase activity was evaluated according to the instructions of the luciferase assay kit (K801-200; Biovision, Mountain View, CA, USA). All steps were repeated 3 times.

### RNA-binding protein immunoprecipitation (RIP) assay

RIP assay was conducted using the RIP kit (R&D) to verify the binding relations between miR-124-3p and MALAT1 and between MALAT1 and KLF5. Chondrocytes (1 × 10^7^) were suspended in RIP lysis buffer (Thermo Fisher), upon which lysates were incubated with magnetic bead-coupled Argonaute-2 (ab186733, 1: 50, Abcam) or IgG (ab6709, 1: 1000, Abcam) at 4 °C overnight. The immunoprecipitation was collected, followed by the extraction of protein using protease K (Thermo Fisher) to extract RNA. The enrichment of miR-124-3p, MALAT1, and KLF5 was analyzed by RT-qPCR.

### RNA pull-down assay

The Pierce™ Magnetic RNA–Protein Pull-Down Kit (R&D) was applied for RNA pull-down assay to assess the binding relation between MALAT1 and KLF5. The biotin was used to label the wild-type probe (Bio-MALAT1 probe-WT), the mutant-type probe (Bio-MALAT1 probe-MUT), and the negative control (Bio-control). Then, mouse chondrocytes were lysed using lysis buffer (Thermo Fisher), followed by incubation with biotin-labeled RNA prober (GenePharma, Shanghai, China). Subsequently, cells were treated with RNase-free DNase I (Thermo Fisher) and subject to RNeasy Mini Kit (R&D) to extract the binding RNA, which was applied for RT-qPCR analysis to detect the abundance of KLF5.

### RNA stability experiment

To evaluate the RNA half-life period of MALAT1, cells in the agomiR-124-3p group and the agomiR-NC group were treated with actinomycin D (2 μg/mL). Total RNA in cells was extracted at 0, 2, 4, 6, and 9 h after actinomycin D treatment. MALAT1 expression at the different time points after actinomycin D treatment was detected by RT-qPCR, with GAPDH serving as the internal reference. Each experiment was conducted 3 times.

### Statistical analysis

SPSS 21.0 software (IBM Corp. Armonk, NY, USA) was appointed for data analysis, and GraphPad Prism 8.0 software (GraphPad Software Inc., San Diego, CA, USA) was used for graphing. The results were presented as mean ± standard deviation. All data were inspected with normality distribution and homogeneity test of variance. The Student’s *t*-test was appointed for comparison analysis between two groups, and one-way or two-way analysis of variance (ANOVA) was appointed for comparison analysis among multiple groups, and Tukey's multiple comparisons test was appointed for the post-test of data. The *p* value was attained using a two-tailed test, a value of *p* < 0.05 indicated a statistical significance, and a value of *P* < 0.01 indicated a highly statistical significance.

## Results

### miR-124-3p overexpression alleviates OA mouse cartilage injury

**Fig. 1:**
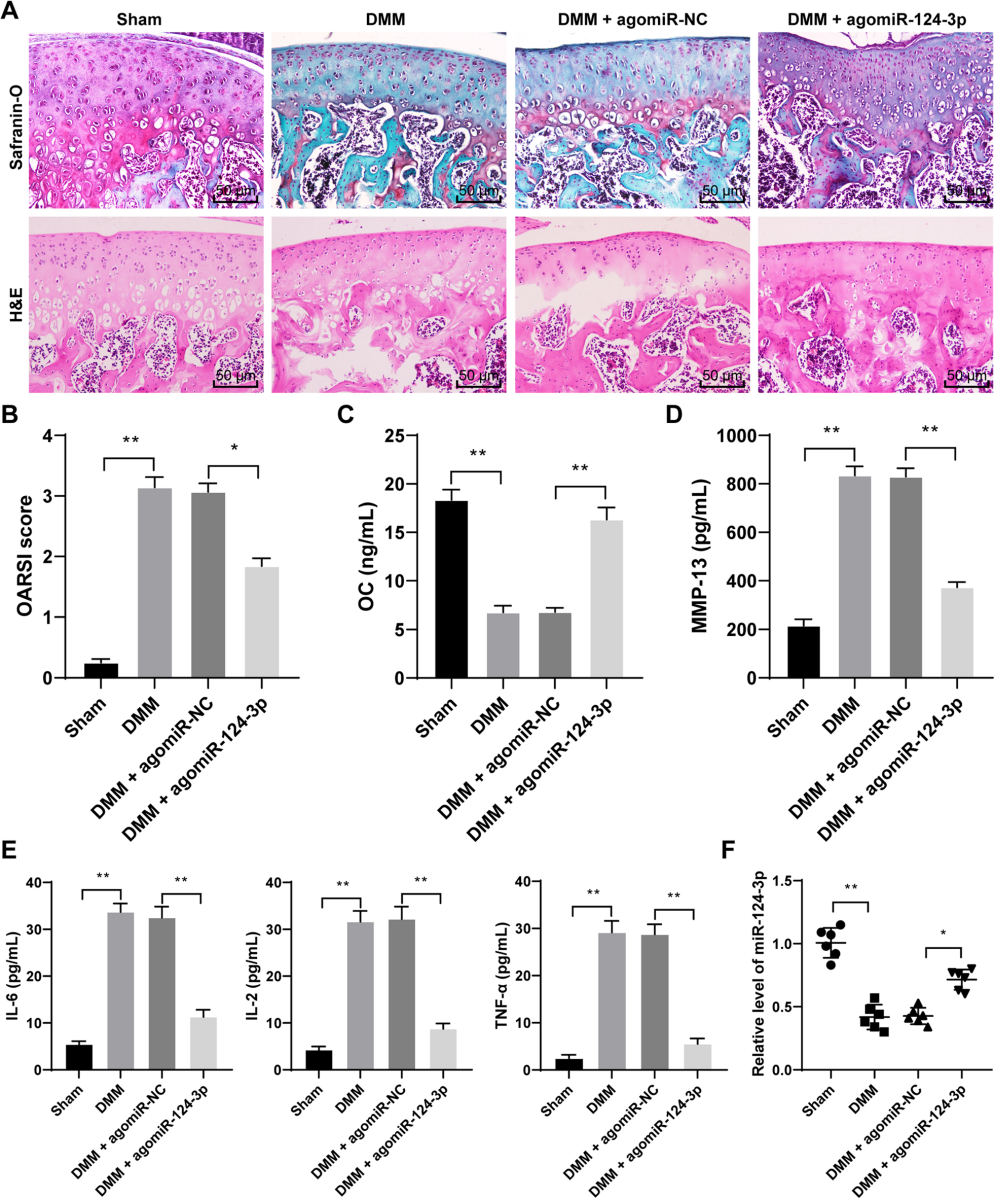
miR-124-3p overexpression alleviates OA mouse cartilage injury. DMM mouse model was established to mimic OA mice, which were injected with agomiR-124-3p to overexpress miR-124-3p, with agomiR-NC as the control. **A**, mouse knee cartilage damage was determined by Safranin-O-fast green staining and H&E staining. **B**, degree of mouse knee cartilage damage was evaluated by OARSI grading. **C**, **D**, and **E**, contents of OC, MMP-13, IL-6, IL-2, and TNF-α in mouse serum were detected by ELISA. **F**, miR-124-3p expression in tissue was measured by RT-qPCR. *N* = 6. The data in panels **B**–**E** were presented as mean ± standard deviation. One-way ANOVA was used to analyze the data in panels **B**, **C**, **D**, **E**, and **F**. Tukey's multiple comparisons test was applied for the post hoc test. *p < 0.05, **p < 0.01

To investigate the regulatory role of miR-124-3p in OA, we established a DMM model to mimic OA mice. The results showed that DMM mice showed obvious OA signs, such as cartilage fiber and cartilage erosion in the knee joint, increased hyaline cartilage (HC) thickness, disorganized articular chondrocytes with a tendency to thicken (Fig. 1A), and elevated OARSI score (*p* < 0.01, Fig. 1B). The OC content in the serum of DMM mice was decreased (*p* < 0.01, Fig. 1C), while the contents of MMP-13, IL-6, IL-2, and TNF-α levels were increased (*p* < 0.01, Fig. 1D, E). In addition, miR-124-3p expression was downregulated in DMM mouse tissue (*p* < 0.01, Fig. 1F). Therefore, miR-124-3p expression was upregulated in vivo via injection of agomiR-124-3p (*p* < 0.05, Fig. 1F). miR-124-3p overexpression alleviated OA symptoms in DMM mice (Fig. 1A), lowered OARSI score (*p* < 0.05, Fig. 1B), increased the OC content in the serum (*p* < 0.01, Fig. 1C), and reduced the levels of, MMP-13, IL-6, IL-2, and TNF-α in the serum (*p* < 0.01, Fig. 1D, E).

### *miR-124-3p overexpression represses mouse chondrocyte pyroptosis *in vivo

**Fig. 2:**
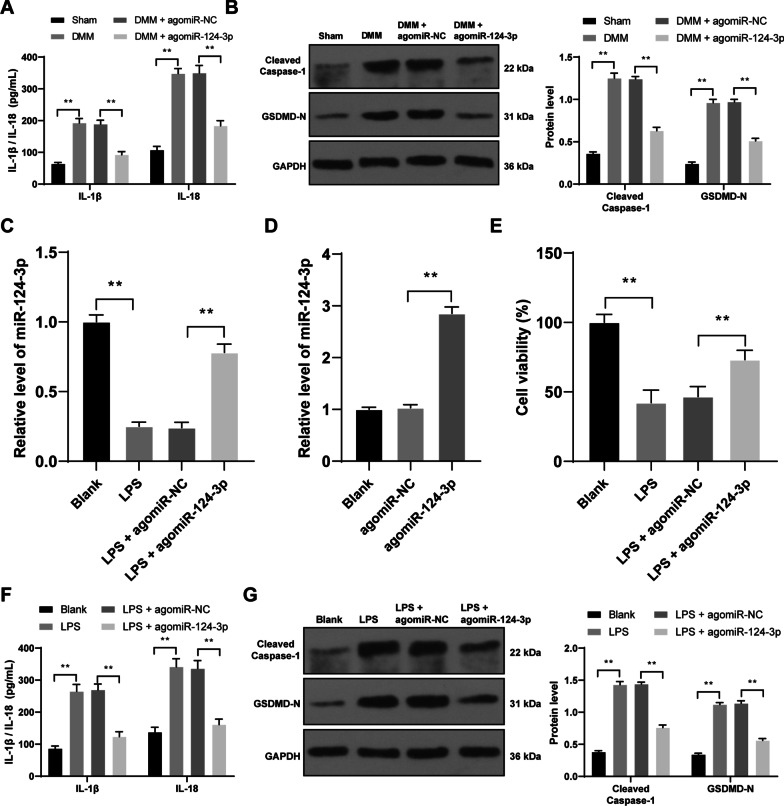
miR-124-3p overexpression represses mouse chondrocyte pyroptosis in vitro and in vivo. agomiR-124-3p was injected into OA mouse knee or transfected into mouse chondrocytes, with agomiR-NC as the control. **A**, expressions of IL‐1β and IL‐18 in the serum were detected by ELISA. **B**, expressions of Cleaved Caspase-1 and GSDMD-N in mouse tissue were detected by western blot analysis. Inflammatory injury in mouse chondrocytes was induced by LPS. **C** and **D**, miR-124-3p expression in chondrocytes (**C**) and miR-124-3p transfection efficiency (**D**) were measured by RT-qPCR. **E**, cell activity was detected by CCK-8 method. **F**, expressions of IL‐1β and IL‐18 in cells were detected by ELISA. **G**, expressions of Cleaved Caspase-1 and GSDMD-N in cells were detected by western blot analysis. *N* = 6, cell experiments were repeated 3 times independently. The results were presented as mean ± standard deviation. Two-way ANOVA was used to analyze the data in panels **A**, **B**, **F**, and **G**, and one-way ANOVA was used to analyze the data in panels **C**, **D**, and **E**. Tukey's multiple comparisons test was applied for the post hoc test. **p < 0.01

Then, we tried to find out the molecular mechanism of pyroptosis in OA cartilage damage. miR-124-3p overexpression declined the levels of IL‐1β, IL‐18, Cleaved Caspase-1, and GSDMD-N in DMM mice (*p* < 0.01, Fig. 2A, B).

Then, lipopolysaccharide (LPS) was employed to induce inflammatory injury in mouse chondrocytes and downregulated miR-124-3p expression in cells (*p* < 0.01, Fig. 2C). Next, miR-124-3p was overexpressed in chondrocytes using agomiR-124-3p (*p* < 0.01, Fig. 2C, D). Compared to the LPS group, the LPS + agomiR-124-3p group had enhanced cell activity (*p* < 0.01, Fig. 2E) and diminished the expressions of IL‐1β, IL‐18, Cleaved Caspase-1, and GSDMD-N (*p* < 0.01, Fig. 2F, G).

### miR-124-3p directly binds to MALAT1 to reduce its stability and expression in OA

**Fig. 3:**
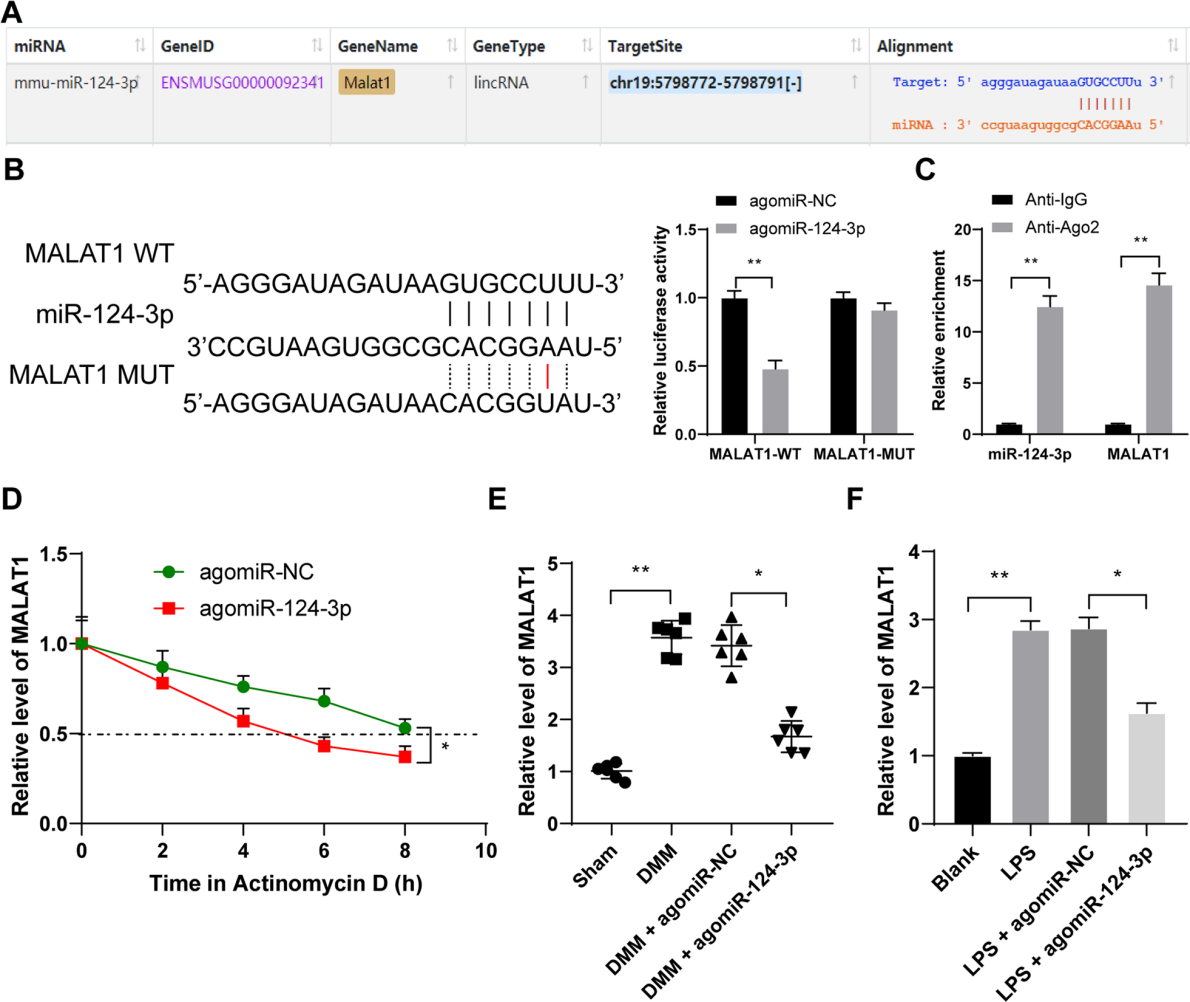
miR-124-3p directly binds to MALAT1 to reduce its stability and expression in OA. **A**, the binding relation between miR-124-3p and MALAT1 was revealed through the StarBase website. **B** and **C**, the binding relation between miR-124-3p and MALAT1 was verified by dual-luciferase reporter gene assay (**B**) and RIP assay (**C**). **D**, half-life period of MALAT1 (as presented by the dotted line) in cells with miR-124-3p overexpression after the treatment of actinomycin **D**. **E** and **F**, MALAT1 expression in tissue (**E**) and chondrocytes (**F**). *N* = 6, cell experiments were repeated 3 times independently. The data in panels **B**–**D**, and **F** were presented as mean ± standard deviation. One-way ANOVA was used to analyze the data in panels **E** and **F**, and two-way ANOVA was used to analyze the data in panels **B**, **C**, and **D**, and one. Tukey's multiple comparisons test was applied for the post hoc test. *p < 0.05, **p < 0.01

The binding relation between miR-124-3p and MALAT1 was revealed through the StarBase website (Fig. 3A), and then, we verified the binding of miR-124-3p to LncRNA MALAT in chondrocytes (*p* < 0.01, Fig. 3B, C), and miR-124-3p quenched MALAT1 stability (*p* < 0.05, Fig. 3D). In addition, MALAT1 expression was robustly upregulated after modeling, and miR-124-3p overexpression reduced MALAT1 expression (*p* < 0.05, Fig. 3E, F).

### MALAT1 overexpression reverses the inhibiting role of miR-124-3p overexpression in chondrocyte pyroptosis

**Fig. 4:**
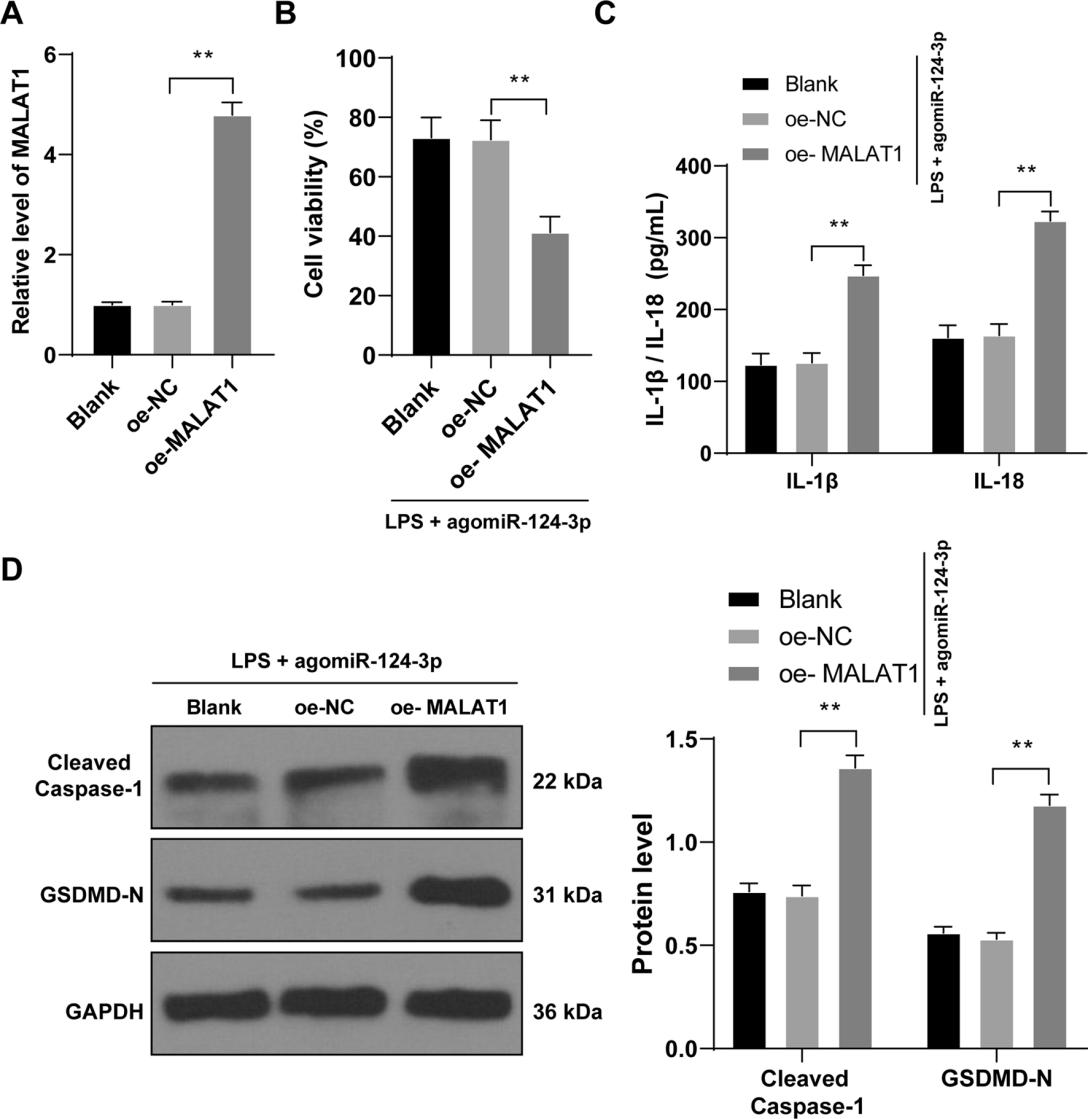
MALAT1 overexpression reverses the inhibiting role of miR-124-3p overexpression in chondrocyte pyroptosis. oe-MALAT1 was transfected into cells to upregulate MALAT1 expression to conduct combined experiments with agomiR-124-3p, with oe-NC transfection as the control. **A**, MALAT1 transfection efficiency was verified by RT-qPCR. **B**, cell activity was detected by CCK-8 method. **C**, expressions of IL‐1β and IL‐18 in mouse cartilage tissue were detected by ELISA. **D**, expressions of Cleaved Caspase-1 and GSDMD-N in mouse cartilage tissue were detected by western blot analysis. Cell experiments were repeated 3 times independently. The results were presented as mean ± standard deviation. One-way ANOVA was used to analyze the data in panels **A** and **B**, and two-way ANOVA was used to analyze the data in panels **C** and **D**. Tukey's multiple comparisons test was applied for the post hoc test. **p < 0.01

To elucidate the effects of MALAT1 on OA chondrocyte pyroptosis, oe-MALAT1 was transfected into cells and successfully overexpressed MALAT1 expression (*p* < 0.01, Fig. 4A), followed by rescue experiments with agomiR-124-3p. Compared with the LPS + agomiR-124-3p group, the LPS + agomiR-124-3p + oe-MALAT1 group showed decreased cell activity (*p* < 0.01, Fig. 4B), elevated IL‐1β and IL‐18 contents (*p* < 0.01, Fig. 4C), and increased expressions of Cleaved Caspase-1 and GSDMD-N (*p* < 0.01, Fig. 4D).

### MALAT1 binds to transcription factor KLF5 to promote CXCL11 transcription

**Fig. 5:**
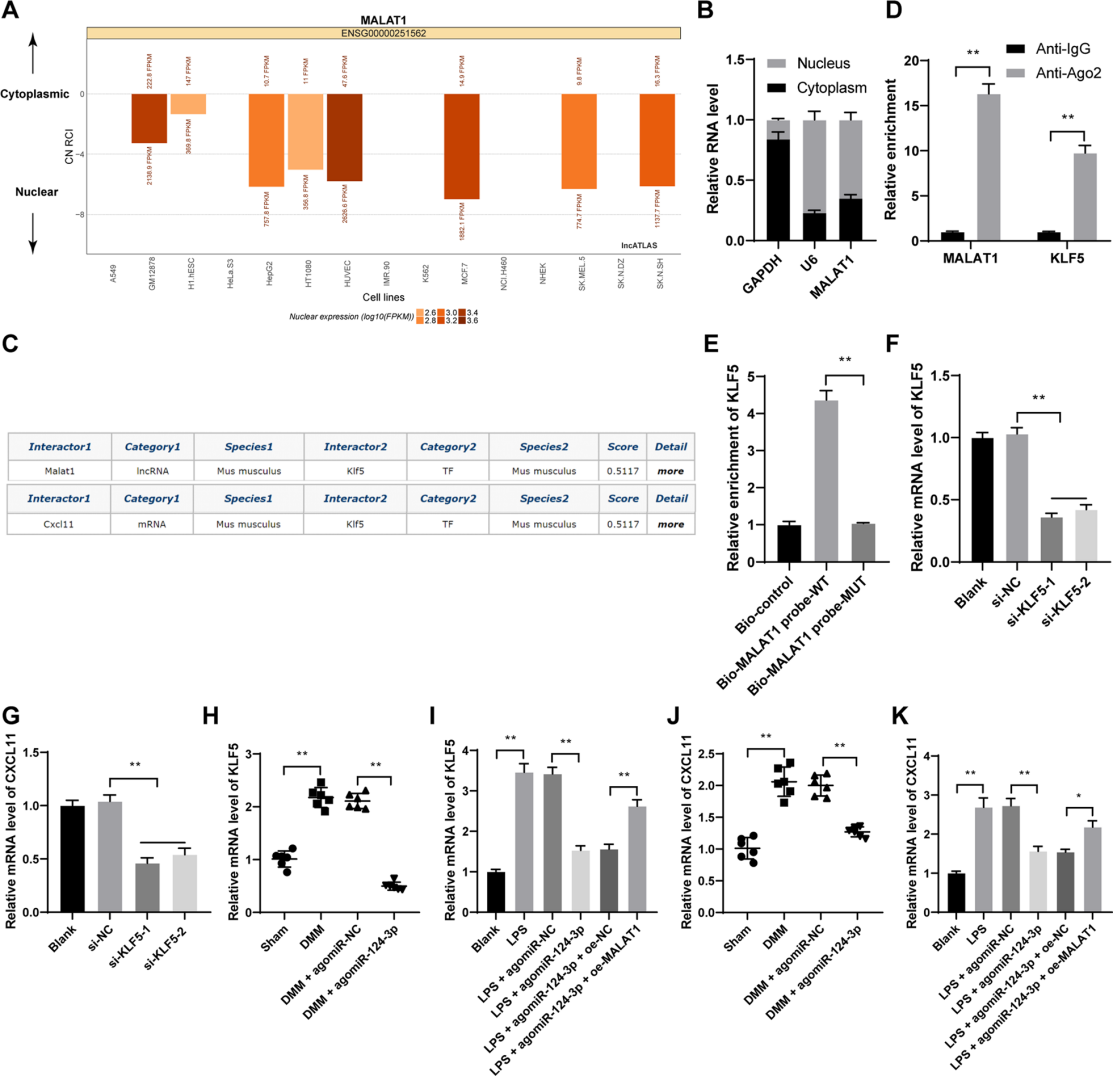
MALAT1 binds to transcription factor KLF5 to promote CXCL11 transcription. **A**, the subcellular localization of MALAT1 was predicted via the LncATLAS website. **B**, fractionation of nuclear and cytoplasmic RNA verified that MALAT1 was mainly localized in the nucleus. **C**, The binding relations between MALAT1 and KLF5 and between KLF5 and CXCL11 were predicted through the RNAInter database. **D** and **E**, the binding relation between KLF5 and MALAT1 was verified by RIP assay (**D**) and RNA pull-down assay (**E**). si-KLF5-1 or si-KLF5-2 was transfected into cells to downregulate KLF5 expression, with si-NC as the control. **F**, KLF5 transfection efficiency was verified by RT-qPCR. **G**, CXCL11 expression in cells with si-KLF5 was detected by RT-qPCR. **H** and **I**, KLF5 expression in tissue (**H**) and chondrocytes (**I**) were determined by RT-qPCR. **J** and **K**, CXCL11 expression in tissue (**J**) and chondrocytes (**K**) were determined by RT-qPCR *N* = 6, cell experiments were repeated 3 times independently. Data in panels **B**, **D**, **E**–**G**, **I**, and **K** were presented as mean ± standard deviation. Two-way ANOVA was used to analyze the data in panel **D**, and one-way ANOVA was used to analyze the data in panels **E**–**K**. Tukey's multiple comparisons test was applied for the post hoc test. *p < 0.05, **p < 0.01

Next, the subcellular localization of MALAT1 was predicted via the LncATLAS website, and it was found that MALAT1 was principally localized in the nucleus (Fig. 5A). In addition, MALAT1 was mainly localized in the nucleus of chondrocytes (Fig. 5B). Via the RNAInter database, the binding relations between MALAT1 and KLF5 and between KLF5 and CXCL11 were predicted (Fig. 5C). Besides, the binding relation between KLF5 and MALAT1 was verified in chondrocytes (*p* < 0.01, Fig. 5D, E). si-KLF5-1 or si-KLF5-2 was transfected into cells and successfully downregulated KLF5 expression (*p* < 0.01, Fig. 5F), and it was found that KLF5 knockdown reduced CXCL11 expression (*p* < 0.05, Fig. 5G). In the DMM and LPS groups, KLF5 and CXCL11 expressions were highly expressed, miR-124-3p overexpression effectively decreased the expression levels of KLF5 and CXCL11, and the decrease was reversed by MALAT1overexpression (*p* < 0.05, Fig. 5H–K).

### CXCL11 overexpression reverses the protective role of miR-124-3p overexpression in chondrocyte pyroptosis

**Fig. 6:**
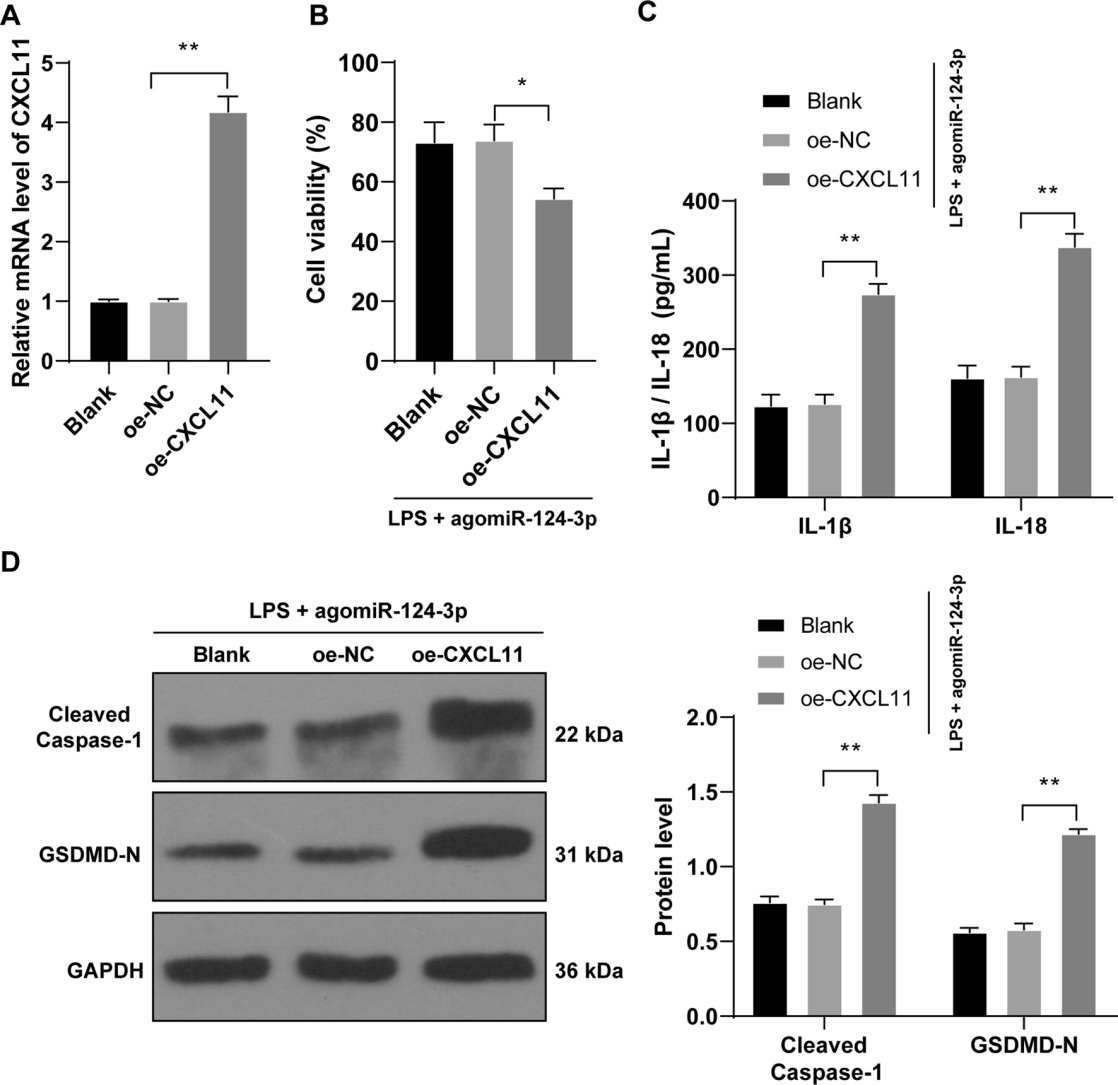
CXCL11 overexpression reverses the inhibiting role of miR-124-3p overexpression in chondrocyte pyroptosis. oe-CXCL11 was transfected into cells to upregulate CXCL11 expression to conduct combined experiments with agomiR-124-3p. **A**, CXCL11 transfection efficiency was verified by RT-qPCR. **B**, cell activity was detected by CCK-8 method. **C**, expressions of IL‐1β and IL‐18 in mouse cartilage tissue were detected by ELISA. **D**, expressions of Cleaved Caspase-1 and GSDMD-N in mouse cartilage tissue were detected by western blot analysis. Cell experiments were repeated 3 times independently. The results were presented as mean ± standard deviation. One-way ANOVA was used to analyze the data in panels **A** and **B**, and two-way ANOVA was used to analyze the data in panels **C** and **D**. Tukey's multiple comparisons test was applied for the post hoc test. *p < 0.05, **p < 0.01

Eventually, we investigated the effects of CXCL11 on OA chondrocyte pyroptosis. oe-CXCL11 was transfected into cells to upregulate CXCL11 (*p* < 0.01, Fig. 6A), followed by rescue experiments with agomiR-124-3p. Compared with the LPS + agomiR-124-3p group, the LPS + agomiR-124-3p + oe-CXCL11 group showed decreased cell activity (*p* < 0.01, Fig. 6B), elevated IL‐1β and IL‐18 contents (*p* < 0.01, Fig. 6C), and increased the expressions of Cleaved Caspase-1 and GSDMD-N (*p* < 0.01, Fig. 6D).

## Discussion

**Fig. 7:**
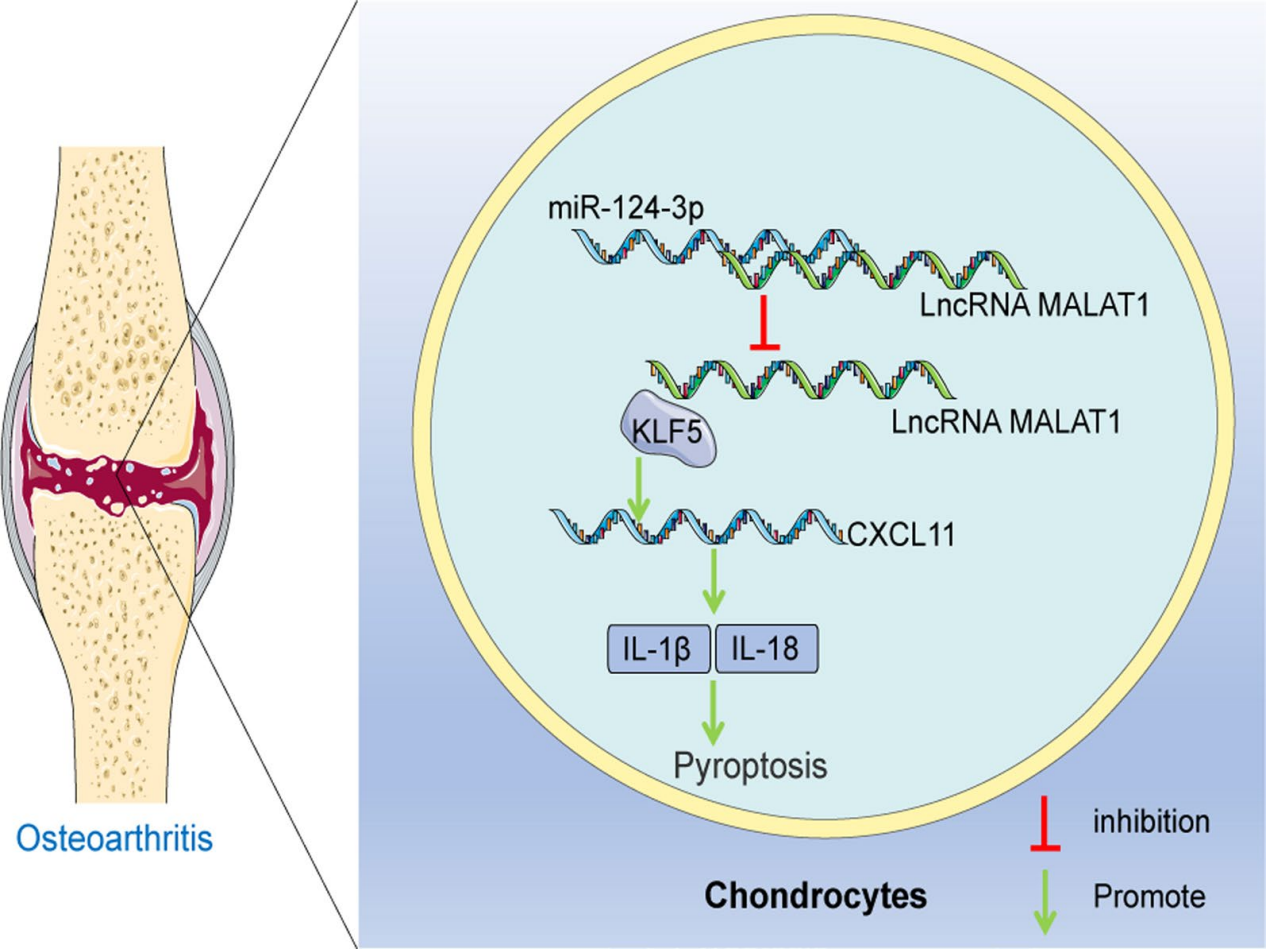
The mechanism of miR-124-3p in OA. miR-124-3p bound to MALAT1 to reduce its stability and expression, repressed the binding of MALAT1 and the transcription factor KLF5, and suppressed CXCL11 transcription, thereby inhibiting chondrocyte pyroptosis and cartilage damage in OA

OA refers to serious cartilaginous disease with high morbidity and leads to mounting health and welfare burdens and loss of functions [26]. Pyroptosis-induced chondrocyte absence and degradation is the principal culprit of inflammatory infiltration and cartilage injury during OA development [27]. Non-coding RNAs, particularly miRNAs, are closely correlated to musculoskeletal injuries in the diseases [8, 28]. miR-124-3p expression is repressed in OA, which is accompanied by joint degradation and dysfunction [11]. In addition, miR-124 affects the expressions of pyroptotic factors to manipulate cell viability and migration [29]. Based on the information, we attempt to discuss the exact mechanism of miR-124-3p in cartilage injury and chondrocyte pyroptosis in OA (Fig. 7).

miRNAs are necessary players and significant indicators of cartilage homeostasis and chondrocyte reproduction in OA as their deficiency exasperates osteal ill-growth and degradation and their involvement regulates molecular progression, cell death, and self-renewal of chondrocytes [30]. miR-124 was weakly expressed in OA, while its expression was reversed with the treatment of a possible medicine [31]. To investigate the role of miR-124-3p in OA, the OA mouse model was established through DMM treatment, and the results unveiled that OA mice showed cartilage fibrosis and erosion in the knee joint, increased HC thickness, disorganized articular chondrocytes with a tendency to thicken, elevated OARSI score, lowered the OC content, increased MMP-13, IL-6, IL-2, and TNF-α levels in the serum, and decreased miR-124-3p expression level in tissue, while these symptoms were all reversed upon agomiR-124-3p treatment. Expectedly, miR-124 overexpression mitigated cartilage tissue damage and encouraged chondrocyte self-renewal [32]. In patients with osteoporosis, OC content was quenched and miR-124-3p was downregulated [33]. Besides, miR-124-3p attenuated inflammatory damages in DMM rats by reducing the contents of IL-6, IL-2, and TNF-α [10]. In summary, miR-124-3p was poorly expressed in OA mice, while miR-124-3p overexpression alleviated cartilage damage in OA mice. Mechanically, as a process resulting from inflammation and related to cellular programmed death, pyroptosis takes an active part in OA pathological progression [34]. miRNAs mediate pyroptotic changes in many human disorders [35]. Although miR-124-3p can render its benign effects on pyroptosis elimination in pulmonary hypertension [12], the mechanism of miR-124-3p in pyroptosis in OA is poorly understood. To find out the molecular mechanism of chondrocyte pyroptosis in OA, LPS was employed to induce inflammatory injury in OA chondrocytes with agomiR-124-3p treatment, after which IL‐1β, IL‐18, Cleaved Caspase-1, and GSDMD-N levels were all diminished. miR-124 can abrogate the inflammatory reactions elicited by LPS [36]. Interestingly, miR-124 upregulation plays a protective role in cerebral ischemia–reperfusion injury by restricting pyroptosis as evidenced by the limited expressions of IL‐1β, IL‐18, Caspase-1, and GSDMD [37]. Collectively, miR-124-3p suppressed chondrocyte pyroptosis in OA.

The regulatory effects of miRNAs on lncRNA transcription and stability in chronic inflammatory conditions have attracted much attention [38]. LncRNAs influence a variety of gene biological behaviors, and they are closely associated with the incidence and severity of OA [39]. The binding of miR-124-3p and MALAT1 was verified in this experiment. To confirm the effects of miR-124-3p on MALAT1 stabilization, cells with miR-124-3p overexpression were treated with actinomycin D, upon which the half-life period of MALAT1 was inhibited. Importantly, a recent finding demonstrated that miR-124-3p and MALAT1 were negatively correlated, and miR-124-3p inhibited MALAT1 expression [40]. The above data demonstrated that miR-124-3p bounds to MALAT1 to reduce its stability and expression.

MALAT1 expression was aggregated in OA, which quenched cell viability and enhanced cartilage injury [41]. The pyroptosis property of lncRNAs is a crucial mechanism in the research and treatment of several diseases [42]. To further elucidate the effects of MALAT1 on OA chondrocyte pyroptosis, oe-MALAT1 was transfected into LPS-induced cells to overexpress MALAT1 expression, and to conduct rescue experiments with agomiR-124-3p. MALAT1 overexpression decreased cell activity, elevated IL‐1β and IL‐18 contents, and increased expressions of Caspase-1 and GSDMD-N. MALAT1 overexpression catalyzed pyroptosis in diabetic nephropathy to accelerate kidney impairment with the involvement of increased IL‐1β, IL‐18, Caspase-1, and GSDMD [43, 44]. Essentially, MALAT1 upregulation could augment the inflammatory injury induced by LPS treatment, to debilitate hindlimb mobility of rodents with acute spinal cord injury [45]. It is plausible that MALAT1 overexpression reversed the protective role of miR-124-3p overexpression in chondrocyte pyroptosis.

Subsequently, we found that MALAT1 could bind to KLF5. It was previously discovered that the binding of miR-124-3p, MALAT1, and KLF5 could modulate pulmonary artery hypertension development, and MALAT1 was positively correlated to KLF5 [46]. Furthermore, KLF5 overexpression was responsible for chondrocyte hypertrophy and inflammatory damage in DMM-triggered OA [47]. Afterwards, the binding of KLF5 and CXCL11 was confirmed. CXCL11 was upregulated in rheumatoid arthritis, with the manifestation of exacerbated inflammatory injury and limited cell activity [48]. To further elucidate the effects of CXCL11 on OA chondrocyte pyroptosis, oe-CXCL11 was transfected into cells with LPS treatment to overexpress CXCL11 and to conduct rescue experiments with agomiR-124-3p. CXCL11 overexpression aggravated pyroptosis of OA chondrocytes. CXCL11 stimulated tissue lesions under an inflammatory microenvironment together with elevated IL‐1β and IL‐18 levels [49]. Additionally, CXCL11 ablation reduced cartilage degradation and inflammatory injury in LPS-induced rheumatoid arthritis [50]. Collectively, CXCL11 overexpression reversed the protective role of miR-124-3p overexpression in chondrocyte pyroptosis.

In conclusion, our findings supported that miR-124-3p bounds to MALAT1 to reduce its stability and expression, repressed the binding of MALAT1 and the transcription factor KLF5 and suppressed CXCL11 transcription, thereby inhibiting chondrocyte pyroptosis and cartilage damage in OA. These findings hinted at a therapeutic strategy for OA alleviation. However, our research just explored the mechanism at the cellular level. In the future, we will further work out to probe the effects of miR-124-3p/MALAT1/KLF5/CXCL11 axis on chondrocyte pyroptosis and cartilage damage in OA through animal experiments.

## Data Availability

The datasets generated during and/or analyzed during the current study are available from the corresponding author on reasonable request.
